# Long-term treatment outcomes with zygomatic implants: a systematic review and meta-analysis

**DOI:** 10.1186/s40729-023-00479-x

**Published:** 2023-07-05

**Authors:** Matthew Brennand Roper, Arjan Vissink, Tom Dudding, Alex Pollard, Barzi Gareb, Chantal Malevez, Thomas Balshi, Lawrence Brecht, Vinay Kumar, Yiqun Wu, Ronald Jung

**Affiliations:** 1 Department of Restorative Dentistry, University Hospitals Bristol and Weston Foundation Trust, Bristol Dental Hospital, Lower Maudlin Street, Bristol, BS1 2LY UK; 2 Department of Oral and Maxillofacial Surgery, Universtitair Medisch Centrum Gronigen, Groningen, The Netherlands; 3 Department of Oral and Maxillofacial Surgery, Clinique Saint-Jean, Brussels, Belgium; 4 PI Dental Center, Fort Washington, USA; 5 Division of Prosthodontics and Restorative Dentistry, NYC College of Dentistry, New York City, NY USA; 6 Department of Oral and Maxillofacial Surgery, Uppsala University, Uppsala, Sweden; 7 Department of Oral Implantology, Second Dental Center, Ninth People’s Hospital Affiliated with Shanghai Jaio Tong University, School of Medicine, Shanghai, China; 8 Clinic of Reconstructive Dentistry, Center of Dental Medicine, University of Zurich, Zurich, Switzerland

**Keywords:** Zygoma, Implant, Atrophic maxilla, Survival, Rehabilitation, Sinusitis, Patient reported outcome

## Abstract

**Purpose:**

The purpose of this study was to perform a systematic review with meta-analysis on the long-term survival rates of zygomatic implants (ZI). ZI success, prostheses survival and success, sinus pathology and patient reported outcomes were also investigated.

**Methods:**

Preferred Reporting Items for Systematic Review and Meta-Analyses (PRISMA) guidelines were followed. Embase and OvidMedline databases were searched alongside the grey literature. The systematic review was recorded in PROSPERO (CRD42022358024). Studies reporting titanium/titanium alloy ZI survival data, ZI-supported prosthesis data, ZIs directly compared to any other implant therapy including grafted sites, a minimum follow-up time of 3 years and a minimum number of 10 patients were included. All study designs were considered if they met the inclusion criteria. Studies not involving ZIs, ZIs not made from titanium/titanium alloy, a follow-up time of < 3 years or < 10 patients, animal studies and in vitro studies were excluded. Long-term follow-up has not been defined in the literature. A minimum of 3 years follow-up was considered acceptable to capture survival after initial healing, alongside in-function prosthesis data via delayed or immediate load protocols. ZI success, was predominantly defined as ZI survival without biological or neurological complications. Meta-analyses were performed for ZI survival, ZI failure incidence, ZI success, loading protocol, prosthesis survival, and prevalence of sinusitis using random effects models. Descriptive analysis was used for ZI success, prosthesis success and patient reported outcome measures.

**Results:**

Five hundred and seventy-four titles were identified, of which 18 met the inclusion criteria. Eligible studies included 1349 ZIs in 623 patients. Mean follow-up period was 75.4 months (range 36–141.6). The mean survival of ZIs was 96.2% [95% CI: 93.8; 97.7] at 6 years. Mean survival for delayed loading was 95% [95% CI: 91.7; 97.1] and 98.1% [95% CI: 96.2; 99.0] for immediate loading (*p* = 0.03). Annual incidence rate of ZI failure was 0.7% [95% CI 0.4; 1.0]. Mean ZI success was 95.7% [95% CI 87.8; 98.6]. Mean prosthesis survival was 94% [95% CI 88.6; 96.9]. Sinusitis prevalence was 14.2% [95% CI 8.8; 22.0] at 5 years. Patients’ reported increased satisfaction with ZIs.

**Conclusions:**

ZIs have long-term survival comparable to conventional implants. Immediate loading showed a statistically significant increase in survival over delayed loading. Prosthesis survival was similar to that of prostheses supported by conventional implants, with similar complications. Sinusitis was the most frequently encountered biological complication. Patients reported improved outcome measures with ZI use.

**Graphical Abstract:**

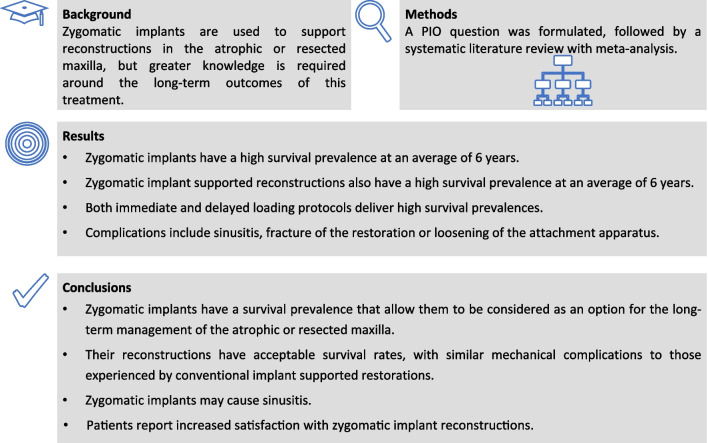

**Supplementary Information:**

The online version contains supplementary material available at 10.1186/s40729-023-00479-x.

## Introduction

Implant-supported rehabilitation of the maxilla represents a significant surgical challenge when bone volumes are inadequate to allow placement of conventional implants with various treatment modalities explored [1]. Patients face the likelihood of protracted treatment times with significant morbidity related to donor harvest sites, and the additional costs related to the use of biomaterials. Prosthodontic rehabilitation may also be delayed, as two-stage procedures are required if primary stability is not achieved, or when hard tissue reconstruction is required prior to implant placement. These challenges may act as barriers to the provision of care, which may otherwise improve the quality of patients’ lives.

The use of the zygoma as an anchorage site was first explored by Branemark in 1988, using customised, increased-length conventional implants. This technique was first reported in the literature by Aparicio [2], when stabilising a graft in the pre-maxilla using the zygomatic process of the maxilla for anchorage. These implants may present an opportunity to bypass the conventional route of hard tissue regeneration, as well as to reduce treatment time frames [3]. Zygomatic implant (ZI) designs are also evolving to meet increased demand in the face of an expanding range of clinical indications, such as oncologic resections or trauma, with key roles including the retention of obturators or in combination with free flap reconstructions [4].

Treatment modalities include ZIs splinted to conventional implants in the anterior maxilla; or the use of a quad zygoma approach [5] for fixed or removable reconstructions, initial prosthetic rehabilitation followed a two-stage approach, but immediate loading has been identified as a viable treatment modality too [6, 7], with some studies reporting this as the preferred protocol in terms of survival outcomes. The anterior–posterior (AP) spread for prosthetic reconstruction can also improved in the case of significantly pneumatised sinuses extending into the canine region, when options are limited for tilted implants [8]. The ability to deliver implants without the conventional challenges as described above, alongside immediate loading, presented a significant step forwards in terms of patient experience and expediency of care in compromised clinical situations. ZIs appear to show good survival rates in the short to medium term [9], however, complications include sinus pathology and oro-antral communications [10, 11]. Prostheses supported by ZIs also appear to show high survival in the short [12] and medium term [13]. There is little data on the long-term survival and success of the prosthetic reconstructions supported by ZIs, or on patient reported outcomes. Therefore, the aim of this systematic review was to assess long-term ZI survival rates, and to report on biological, prosthetic, mechanical and patient reported outcomes (PROMS) based on previously published clinical studies. The null hypothesis was no difference in the prevalence of ZI survival, or the prevalence and complications related to ZI-supported reconstructions, when compared to conventional implants and their reconstructions in the maxilla.

## Methods

Preferred Reporting Items for Systematic Review and Meta-Analyses (PRISMA) guidelines were followed for this review. The systematic review was recorded in the International Prospective Register of Systematic Reviews (PROSPERO) under registration number CRD42022358024.

### Eligibility criteria

Inclusion criteria were formulated using the PIO format. The population (P) included adults over the age of 18, who had received titanium/titanium alloy ZIs. The intervention (I) was the use of restored and unrestored ZIs. The outcome (O) was ZI survival with secondary outcomes including ZI success, prosthetic survival and success, prosthetic complications, sinus pathology and patient reported outcomes over a minimum of 3 years follow-up.

### Inclusion criteria

Clinical studies which met the following inclusion criteria were included: Studies including ZIs, studies including ZI-supported fixed or removable reconstructions, ZIs made from titanium/titanium alloy, studies directly comparing ZIs to any other conventional implant therapy (included grafted sites), a minimum follow-up time of 3 years and a minimum number of 10 patients. Study designs included were randomised controlled trials, clinical trials, prospective case series and retrospective case series.

### Exclusion criteria

The exclusion criteria were clinical studies that did not involve ZIs, ZIs not made from titanium/titanium alloy, a follow-up time of less than 3 years, or with less than 10 patients included. Animal studies and in vitro studies were excluded.

### Study identification

Studies were extracted from Embase, dating 1974 to June 21, 2022 and from Ovid MEDLINE dating 1946 to June 21 2022. The full search strategy table is tabulated in Additional file 1: Table S1. The search was not limited by language.

### Outcome measures

ZI survival (presence or absent at follow-up) was the primary outcome measure. Secondary outcomes included ZI success, ZI-supported prosthesis survival, success and complications, sinus pathology and patient reported outcomes. Heterogeneity was noted in the reporting of ZI success, which was predominantly defined as ZI survival without biological or neurological complications across the studies. 

### Data extraction

Data were independently extracted and assessed by two reviewers (MBR and TD). Collection included authors, year of publication, patient data, outcomes reported, loading protocols and follow-up periods. Outcomes recorded were ZI survival, ZI success, ZI-supported prosthesis survival and/or success, sinus data and PROMs. Study authors were contacted in the event of missing data and the report excluded in the event of no reply or inadequate data.

### Risk of bias assessment

The papers included were case series reports. Therefore, the critical appraisal checklist for case series, developed by the Joanna Briggs Institute (critical appraisal tools for use in systematic reviews, 2017) was used to assess the risk of bias. This checklist identified the completeness of the report, risk of bias, and the accuracy of reporting. Reports were independently assessed by two reviewers (MBR and AP) with conflicting outcomes resolved by discussion with a third reviewer (TD).

### Statistical analysis

Effect measures were generated for ZI survival prevalence (%), ZI failure incidence rate (%/year), ZI success prevalence (%), prosthesis survival prevalence (%), and sinusitis prevalence (%). ZI failure incidence rate was calculated as the total annual failure incidence rate over the complete follow-up (%/year), failure incidence in the first year (%), and annual failure incidence after the first year of follow-up (%). In addition, an a priori specified subgroup analysis comparing the ZI survival (%) between the different loading protocols (immediate versus delayed) was performed. Statistical heterogeneity was assessed as substantial if *I*^2^ was > 50% [14]. All meta-analyses were performed in R (v4.2.2, *meta*-package) using a random effects model with the DerSimonian–Laird estimator. Reporting bias was assessed through funnel plots if > 10 studies were available per endpoint if no clinical or statistical heterogeneity was observed. In all analyses, *p* < 0.05 (two-tailed) was considered statistically significant.

## Results

### Selection process

**Fig. 1:**
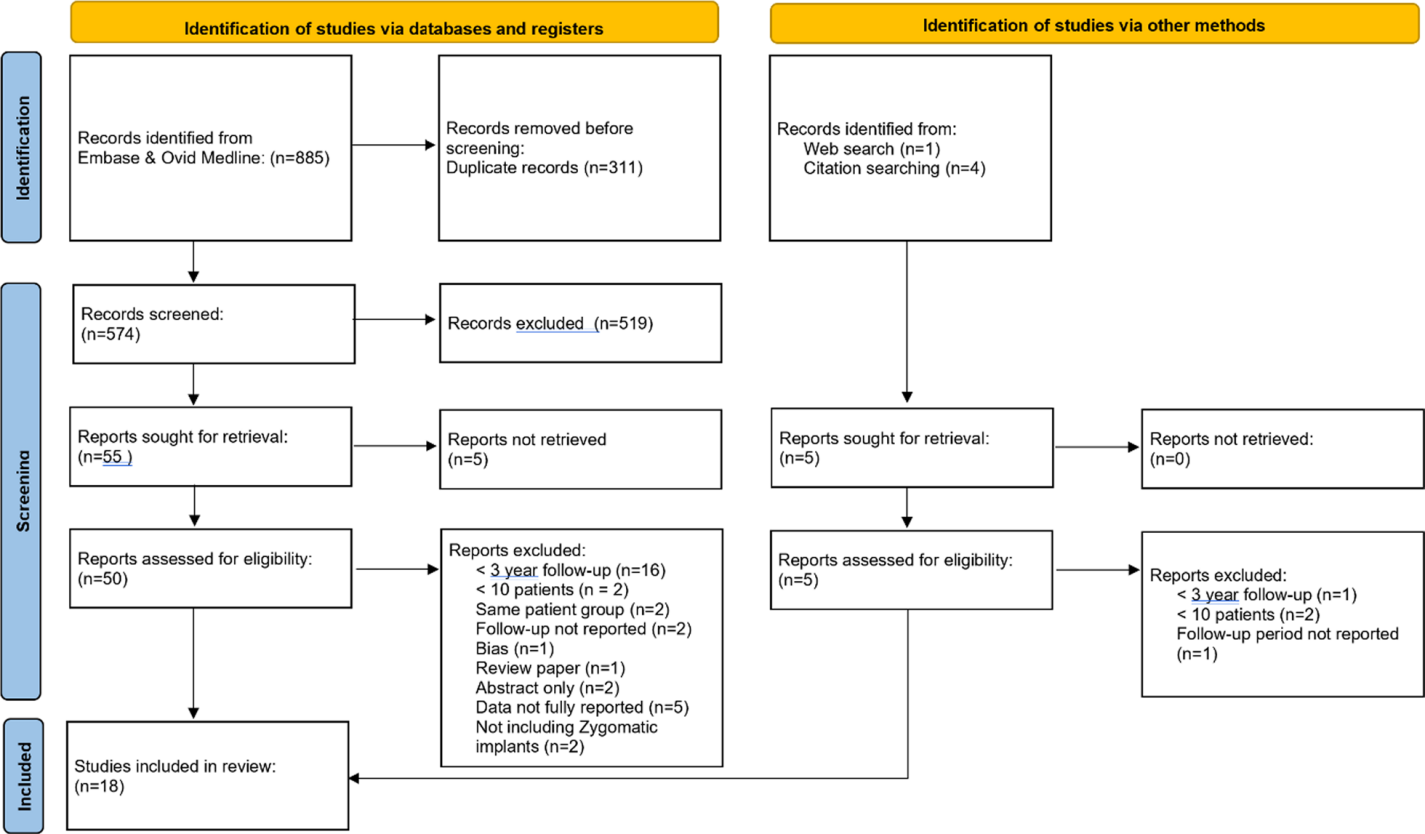
Flow diagram of study identification process

An initial screening of 574 titles and abstracts was carried out by the main author (MBR) (Fig. 1). The grey literature search identified 5 studies. 55 papers were subsequently sought for retrieval. Full-text articles were not retrieved for 5 reports leaving 50 reports to be assessed for eligibility.

These reports were independently reviewed according to the inclusion and exclusion criteria by two reviewers (MBR and TD). The studies included, along with the extracted data, are presented in Table 1, whilst patient demographics are presented in Table 2.

**Table 1:** Datasets extracted from the included studies (*n* = 18)

Study	Year of publication	Number of patients	Mean follow-up (months)	Outcomes reported	Loading protocol	Study type	Zygomatic implant number	Female	Mean age	Age range	Smokers
Kahnberg et al. 2007	2007	76	36	Survival Prosthesis success Sinus data PROM	Delayed	Prospective Case Series	145	57	58	35–77	15
Pellegrino et al. 2020	2020	20	39.9	Survival Success Prosthesis survival Prosthesis success PROM	Immediate	Prospective Case Series	73	NR	56.7	± 12.6	9
Aparicio et al. 2014	2014	80	55.44	Survival Sinus data PROM	Delayed	Retrospective Case Series	157	55	3.81	42.78–64.78	24
Coppede et al. 2017	2017	42	60	Survival	Immediate and delayed	Prospective Case Series	94	32	58	37–79	8
Davo and Pons 2015	2015	14	60	Survival Success Prosthesis survival Sinus data PROM	Immediate	Prospective Case Series	68	10	57.7	41–78	NR
Malo et al. 2014	2014	39	60	Survival Success Prosthesis survival Sinus data PROM	Immediate	Retrospective Case Series	92	30	53.5	32–77	4
Davo, Malevez and Pons 2013	2013	36	60	Survival Success Prosthesis success Sinus data	Immediate	Prospective Case Series	69	23	57.5	34–79	NR
Davo 2009	2009	21	60	Survival Prosthesis success Sinus data	Delayed	Retrospective Case Series	39	16	51.4	36–72	NR
Branemark et al. 2004	2004	28	60	Survival Prosthesis survival Sinus data	Delayed	Prospective Case Series	52	16	58.3	39–79	NR
Yates et al. 2013	2013	23	72	Survival Success	Delayed	Retrospective Case Series	43	13	NR	NR	6
Bedrossian E. 2010	2010	36	84	Survival	Immediate	Prospective Case Series	74	22	NR	NR	NR
Agliardi et al. 2017	2017	15	85.04	Survival Success Prosthesis success Sinus data PROM	Immediate	Prospective Case Series	42	13	62	46–70	NR
Chana et al. 2019	2019	45	90	Survival Prosthesis survival Sinus data	Immediate and delayed	Retrospective Case Series	88	23	56.27	± 13	14
Miglioranca et al*.* 2012	2012	21	96	Survival Prosthesis survival	Immediate	Prospective Case Series	40	13	55	± 6.66	14
Fortin 2017	2017	58	100.8	Survival Success	Immediate and delayed	Retrospective Case Series	107	40	65.3	± 8	NR
Bothur et al*.* 2015	2015	14	111.6	Survival Prosthesis survival	Delayed	Prospective Case Series	58	9	60	51–78	3
Aparicio et al. 2014	2014	22	120	Survival Prosthesis survival Sinus data PROM	Delayed	Prospective Case Series	41	14	63.10	48–80	5
Di Cosola et al. 2021	2021	33	141.6	Survival Success Sinus data	Immediate and delayed	Retrospective case series	67	17	59.1	NR	7

**Table 2:** Included studies and characteristics (*n* = 18)

Study	Study type	Zygomatic implant number	Female	Mean age	Age range	Smokers
Kahnberg et al. 2007	Prospective case series	145	57	58	35–77	15
Pellegrino et al. 2020	Prospective case series	73	NR	56.7	± 12.55	9
Aparicio et al. 2014	Retrospective case series	157	55	3.81	42.78–64.78	24
Coppede et al. 2017	Prospective case series	94	32	58	37–79	8
Davo and Pons 2015	Prospective case series	68	10	57.7	41–78	NR
Malo et al. 2014	Retrospective case series	92	30	53.5	32–77	4
Davo, Malevez and Pons 2013	Prospective case series	69	23	57.5	34–79	NR
Davo 2009	Retrospective case series	39	16	51.4	36–72	NR
Branemark et al. 2004	Prospective case series	52	16	58.3	39–79	NR
Yates et al. 2013	Retrospective case series	43	13	NR	NR	6
Bedrossian 2010	Prospective case series	74	22	NR	NR	NR
Agliardi et al. 2017	Prospective case series	42	13	62	46–70	NR
Chana et al. 2019	Retrospective case series	88	23	56.27	± 12.95	14
Miglioranca et al*.* 2012	Prospective case series	40	13	55	± 6.66	14
Fortin 2017	Retrospective case series	107	40	65.3	± 8	NR
Bothur et al*.* 2015	Prospective case series	58	9	60	51–78	3
Aparicio et al. 2014	Prospective case series	41	14	63.10	48–80	5
Di Cosola et al. 2021	Retrospective case series	67	17	59.1	NR	7

*NR* not reported

Excluded studies and the reasons for exclusion are presented in Additional file 1: Table S2.

### Zygomatic implant survival

#### Survival description across studies

Eligible studies included a total of 1349 ZIs placed in 623 patients. Survival was determined by the presence or absence of a ZI at completion of the study. The mean follow-up period across all studies was 75.4 months (6.3 years) with a follow-up range of 36 to 141.6 months (3 years–11.8 years). Table 3 reports the ZI survival dataset.

**Table 3:** Zygomatic implant survival dataset

Study	Patient number	Zygomatic implant number	Number of failed implants	Number of patients failure in	Survival	Follow-up Mean (months)	Follow-up range (months)
Kahnberg et al. 2007	76	145	5 (3.45%)	4 (5.26%)	96.5	36	N/A
Pellegrino et al. 2020	20	73	2 (2.75%)	2 (10%)	97.3	39.9	± 19.5
Aparicio et al. 2014	80	157	5 (3.18%)	2 (2.5%)	96.8	55.4	± 234.7
Coppede et al. 2017	42	94	1 (1.06%)	1 (2.38%)	98.9	60	N/A
Davo and Pons 2015	14	68	0 (0.00%)	0 (0.00%)	100	60	N/A
Malo et al. 2014	39	92	1 (1.09%)	1 (2.56%)	98.9	60	N/A
Davo, Malevez and Pons 2013	36	69	1 (1.45%)	1 (2.78%)	98.6	60	N/A
Davo 2009	21	39	1 (2.56%)	1 (4.76%)	97.4	60	N/A
Branemark et al. 2004	28	52	3 (5.77%)	3 (10.71%)	94.2	60	60–120
Yates et al. 2013	23	43	6 (13.95%)	6 (26.09%)	86.1	72	48–72
Bedrossian 2010	36	74	2 (2.70%)	2 (5.56%)	97.3	84	− 84
Agliardi et al. 2017	15	42	0 (0.00%)	0 (0.00%)	100	85	73–91
Chana et al. 2019	45	88	5 (5.69%)	3 (6.66%)	94.3	90	Not reported
Miglioranca et al*.* 2012	21	40	1 (2.50%)	1 (4.76%)	97.5	96	Not reported
Fortin 2017	58	107	0 (0.00%)	0 (0.00%)	100	101	60–156
Bothur et al*.* 2015	14	58	2 (3.45%)	1 (7.14%)	96.6	112	69.6–144
Aparicio et al. 2014	22	41	2 (4.88%)	1 (4.55%)	95.1	120	± 154.1
Di Cosola et al. 2021	33	67	16 (23.88%)	8 (24.24%)	76.1	142	109–198

**Fig. 2:**
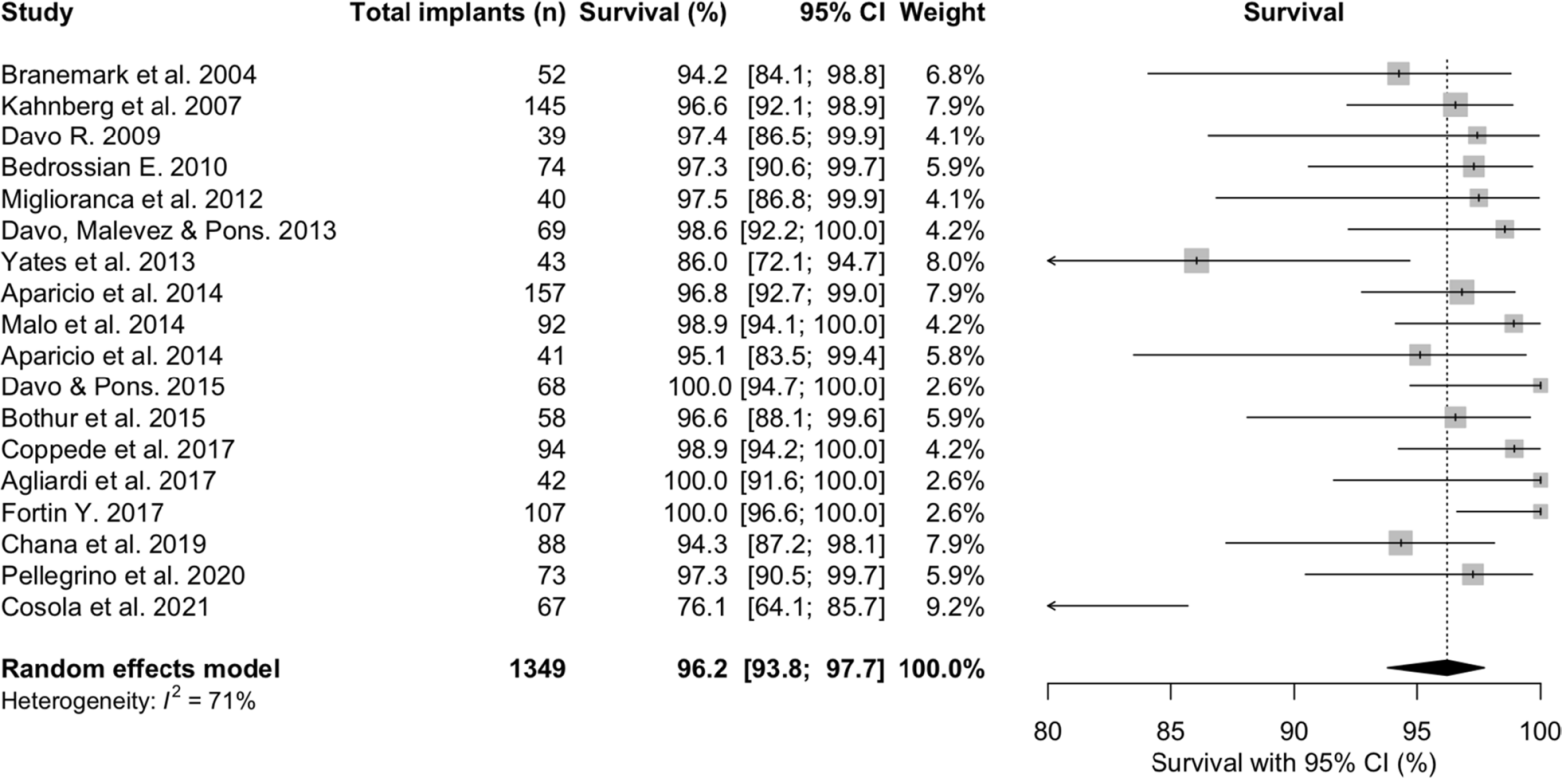
Zygomatic implant survival prevalences at latest follow-up (%)

The mean survival at 75.4 months was 96.2% [95% CI 93.8; 97.7] (Fig. 2). The lowest survival was 76.1% [95% CI 64.1; 85.7] survival at 141.6 months [30], whilst 3 studies reported 100% survival at 60, 85, and 101 months, respectively [13, 18, 27].

**Fig. 3:**
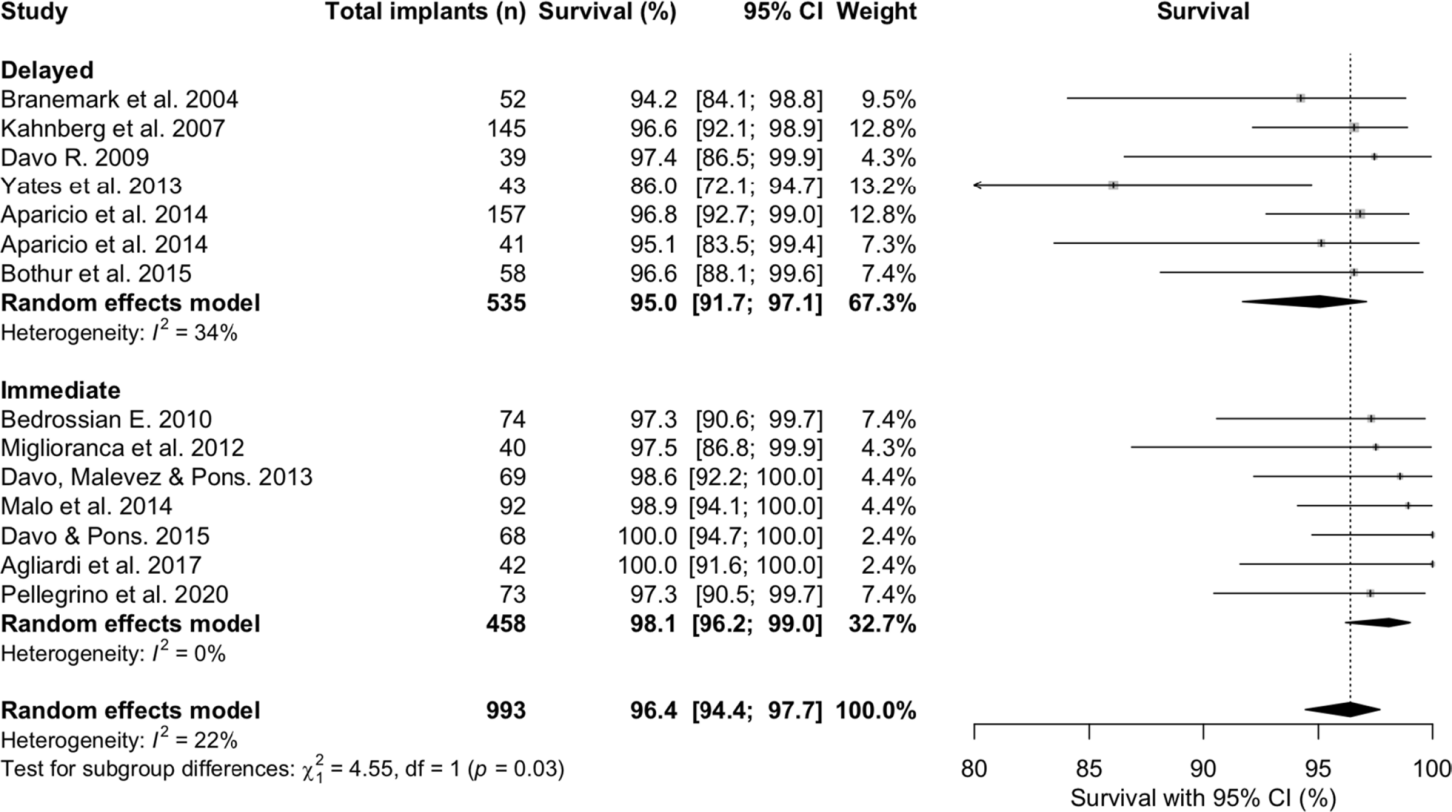
Subgroup analysis comparing the zygomatic implant survival prevalences at latest follow-up between delayed versus immediate loading protocols

When comparing studies with immediate loading to delayed (Fig. 3), mean survival prevalences for delayed load protocols were 95% [95% CI 91.7; 97.1] over a mean of 69.3 months follow-up, and 98.1% [95% CI 96.2; 99.0] over a mean of 73.6 months follow-up for immediate loading protocols (*p* = 0.03).

### Incidence of zygomatic implant failure

**Fig. 4:**
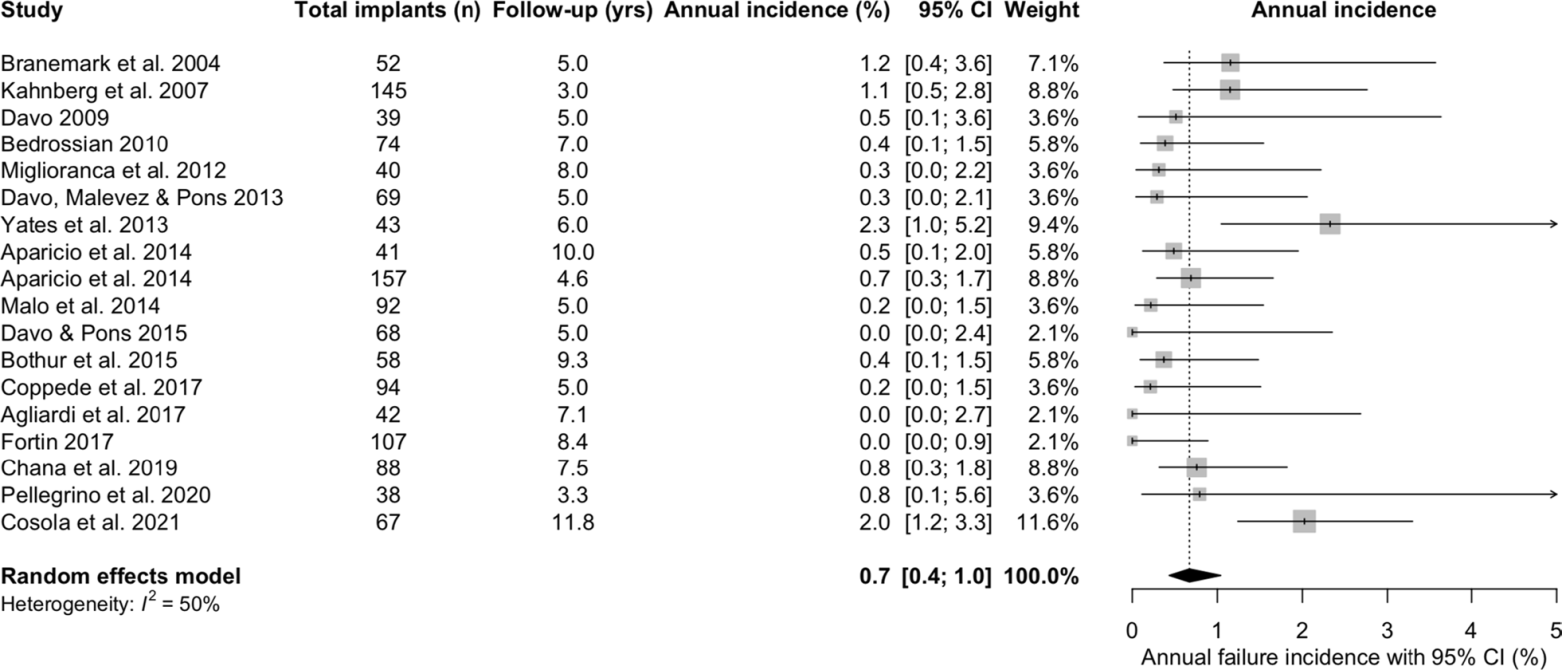
Total annual incidence (%/year) of failure of zygomatic implants

Zygomatic implant failure was defined as the antagonist of ZI survival. The total annual incidence of ZI failure across the studies was 0.7%/year [95% CI 0.4; 1.0] (Fig. 4).

**Fig. 5:**
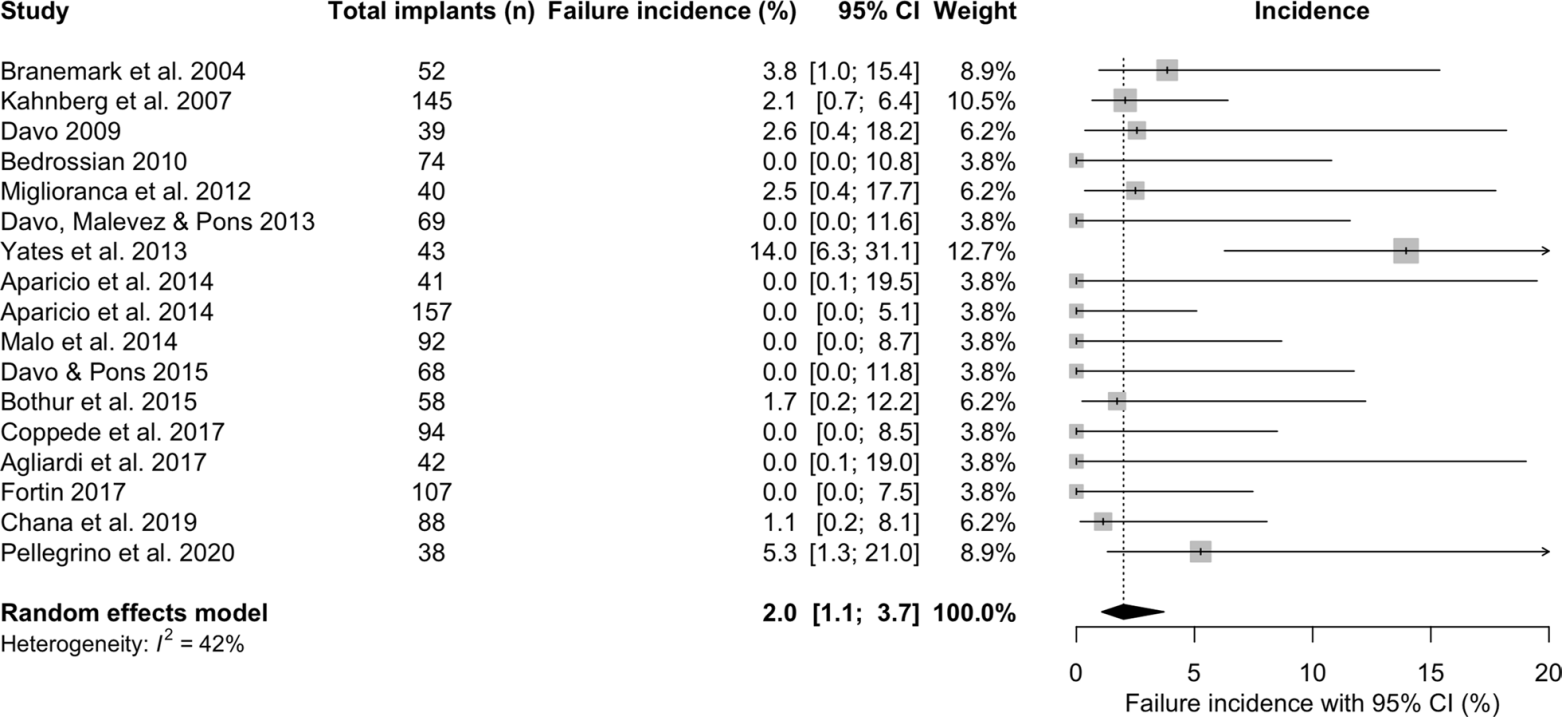
Incidence of zygomatic implant failure within the first year (%)

**Fig. 6:**
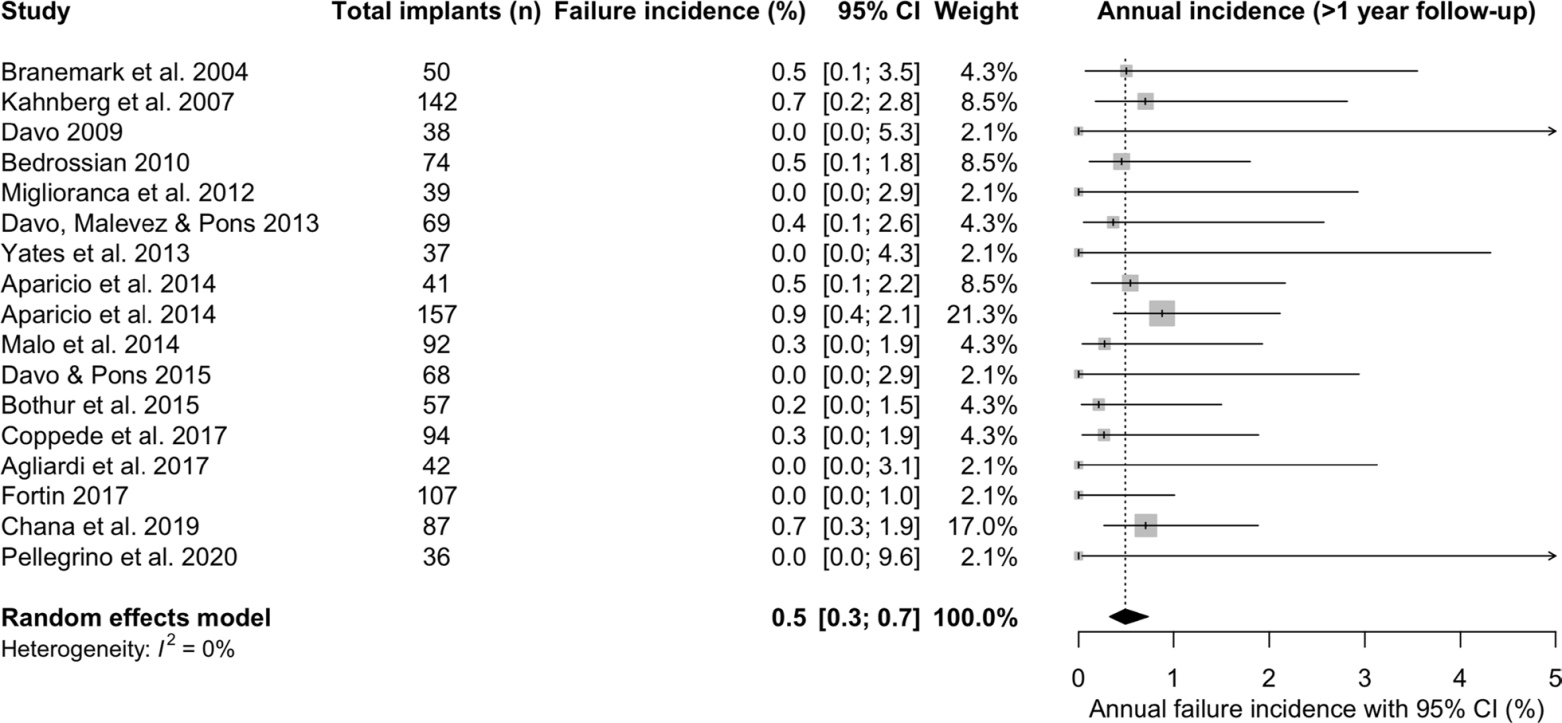
Incidence of zygomatic implant failure after the first year (%)

The incidence of ZI failure within the first year was 2% [95% CI 1.1; 3.7] following placement (Fig. 5), whilst the incidence of ZI failure after the first year following placement was 0.5%/year [95% CI 0.3; 0.7] (Fig. 6)

A significant relationship between implant failure and gender was reported by one study, Di Cosola et al. [30] (males compared with females (*p* < 0.05). No other evaluated conditions (age, smoking, hypertension, or diabetes) were correlated with failure. No statistically significant association between implant failure and sex, surface finish, implant length, or position (*p* > 0.05) [25]. No other studies identified relationships between implant failure and patient demographic.

The classification of ZI placement across the studies was split between the classical Branemark approach (intrasinus), the extra-sinus classification as reported by Miglioranca [26], and the zygomatic anatomy guided approach (ZAGA) developed by Carlos Aparicio [31]. Within this systematic review, no relationship was identified between approaches and survival rates.

Table 4 documents reasons for ZI loss where reported.

**Table 4:** ZI loss reported across studies

Reason attributed to ZI loss	Total number of lost ZIs	Total number of ZIs combined in studies	Study
Sinusitis	19 (12.25%)	155	Di Cosola et al., Chana et al.
Oro-antral communication	1 (1.09%)	92	Malo et al.
Failure of osseointegration	5 (2.34%)	214	Chana et al., Bedrossian et al., Brannemark et al.
Loss of osseointegration	11 (2.39%)	461	Coppede et al., Bothur et al., Yates et al., Davo, Malevez and Pons, kahnberg et al., Branemark et al., Aparicio et al.
Infection or peri-implantitis	4 (3.10%)	129	Yates et al., Aparicio et al.
Pain	3 (2.36%)	127	Chana et al., Davo
Incorrect position	1 (2.32%)	43	Yates et al.
Zygomatic implant fracture	1 (0.64%)	157	Aparicio et al.
Unreported reasons for failure	8 (3.37%)	237	Fortin, Pellegrino et al., Davo and Pons, Agliardi et al.

### Zygomatic implant success

**Fig. 7:**
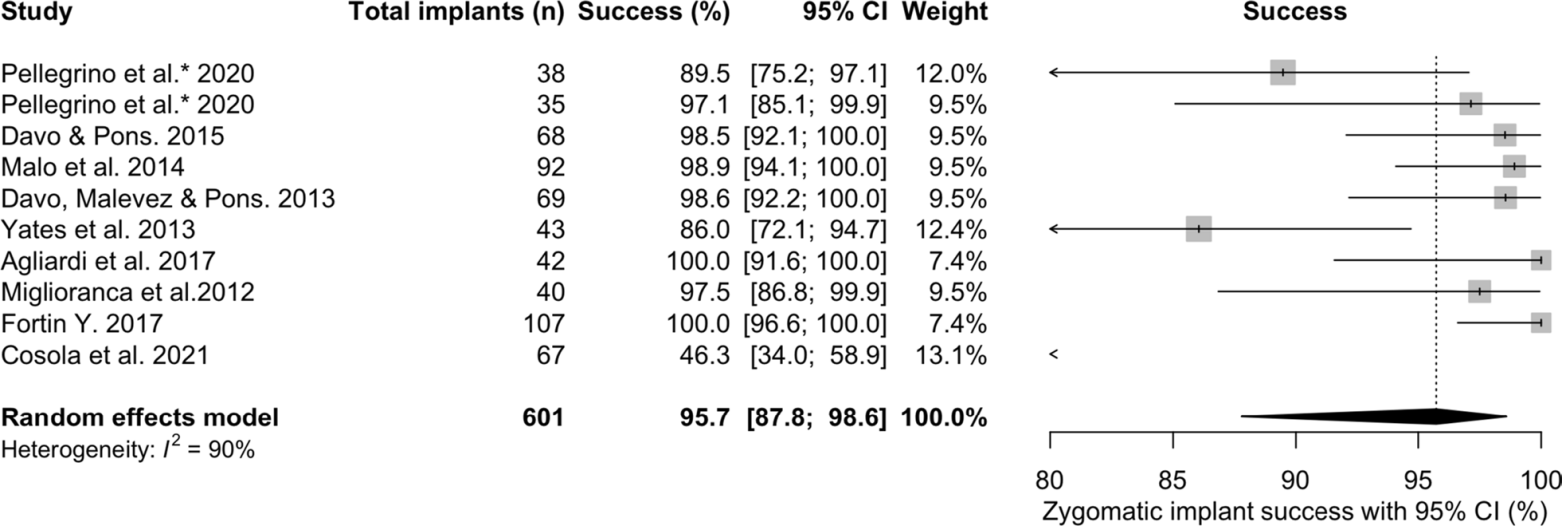
Zygomatic implant success prevalence over the follow-up period

The mean ZI success was 95.7% [95% CI 87.8; 98.6] (Fig. 7) over a mean follow-up of 71.5 months. ZI success was predominantly defined as ZI survival without biological or neurological complications. Success ranged from 46.3% [95% CI 34.0; 58.9] [30] to 100% [95% CI 91.6; 100] [13]. Failure to meet success criteria included unfavourable positioning [18], peri-implant mucositis [16], bleeding on probing and increased pocket depths [19, 25], recession and extra-oral Infective processes [16, 27].

Table 5 reports ZI success data and reasons for failure to meet success criteria across the studies.

**Table 5:** Zygomatic implant success report

Study	Zygomatic implant number	Success	Failure to meet success	Patient number	Follow-up	Follow-up range
Pellegrino et al.^a^ 2020 Atrophic Oncologic	38 35	89.8% (CI: 60.4–97.7%) (*n* = 34) 96.7% (CI:79.2–99.5%) (*n* = 34)	Mucositis: 13.1% (3.7–41%) Mucositis: 39.7% (9.7–91.7%) Extra-oral swelling (*n* = 1)	10 10	39.9	± 19.5
Davo and Pons 2015	68	98.5% (*n* = 67)	Unfavourable position (*n* = 1)	14	60	N/A
Malo et al. 2014	92	98.8% (*n* = 91)	PPD > 4 mm (*n* = 23)	39	60	N/A
Davo, Malevez and Pons. 2013	69	98.55% (*n* = 68)	NR	42	60	N/A
Yates et al. 2013	43	86.05% (*n* = 37)	Recession of 2–4 threads (*n* = 6)	25	72	48–72
Agliardi et al. 2017	42	100% (*n* = 42)	NR	15	85	73–91
Miglioranca et al. 2012	40	97.5% (*n* = 39)	NR	21	96	Not reported
Fortin 2017	107	100% (*n* = 107)	Successful treatment of infection by implant apicoectomy. (not included in success data by study)	58	100.8	60–156
Cosola et al. 2021	67	46.3% (*n* = 36)	28 ZI (41.8%) experienced infective complications defined as sinusitis, oro-antral fistula or soft tissue infection. Early neurologic pain following treatment in 8 ZI (11.9%) in 5 patients	33	141.6	109–198

*NR* not reported

^a^Reported as two groups in the same study

### Zygomatic implant-supported prostheses: s*urvival and success*

**Fig. 8:**
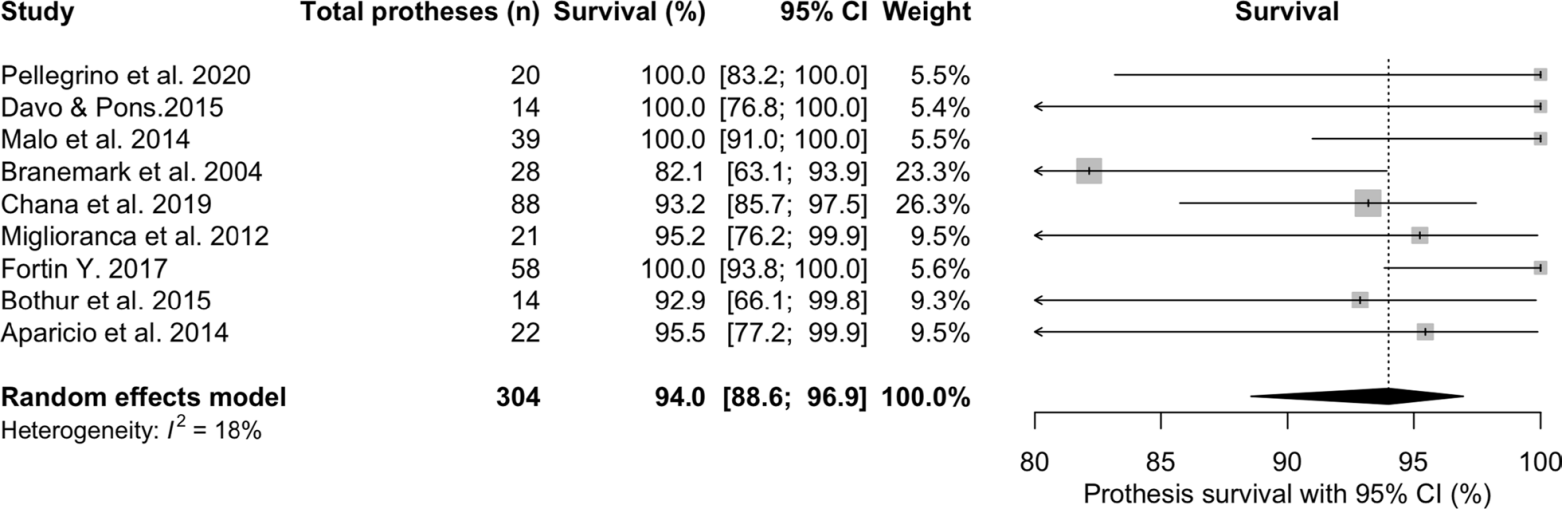
Prosthesis survival prevalence at latest follow-up period (%)

The mean prosthesis survival was 94% [95% CI 88.6; 96.9] at 76 months of mean follow-up (Fig. 8). Seven studies conducted an immediate load protocol, with an interim prosthesis placed immediately after surgery, and was replaced after 3–6 months with the permanent prosthesis. Four conducted both immediate and delayed protocols and 7 conducted delayed protocols.

Table 6 reports prosthesis survival and success data. Survival was determined by the presence or absence of the permanent reconstruction at time of study completion. Success criteria varied across the reports. The main subgroups for failure to meet success included prosthetic tooth loss, chipping or fracture of the veneering material, abutment or screw fracture and abutment or screw loosening.

**Table 6:** Prosthesis survival and success data

Study	Prosthesis number	Prosthesis survival	Prosthesis success	Success criteria	Prosthesis fracture	Prosthetic tooth loss	Screw/abutment loosening/fracture	Follow-up
Kahnberg et al. 2007	60		83.34% (*n* = 50)	Clinically stable prosthesis that not been removed for two weeks or more during the study period		2 cases requiring laboratory repair	1 patient with loosening of ball attachments	36
Pellegrino et al. 2020	20	100% (*n* = 20)	90% (*n* = 9) (95% CI: 47.3–98.5%) Atrophic 58.3% (*n* = 6) (95% CI: 22.9–82%) Oncologic difference between the groups was not statistically significant (*p* = 0.77)	Support of all placed implants, extended to the first molar region, not need to be reduced from the day of delivery to the final follow-up			Prosthetic screw loosening and abutment fractures. All unsuccessful prosthetic events occurred in the first year, with the majority experienced by the oncologic group. 5-year follow-up complication probability values were 10.7% [95% CI 4.1–26%] for the atrophic group, and 10.7% [95% CI 3.5–29.6%] for the oncologic group	39.9
Aparicio et al. 2014	80	NR	NR	NR	65 events involving fracture of the prosthetic acrylic coating material occurred. 2 fractures of the porcelain coating occurred. No metal framework fractures	NR	16 screws and abutments loosened within the ZAGA group, and 7 screws fractured	55.44
Davo and Pons 2015	14	100% (*n* = 14)		Clinically stable prosthesis that not been removed for two weeks or more during the study period	1 incidence of acrylic fracture from the underlying substructure at 36 months	1 incident involving loss of an anterior tooth from an acrylic prothesis	Fracture of 1 abutment at 12 months	60
Coppede et al. 2017	42	NR	NR	NR	NR	Fractures or detachments in 5 fixed implant-supported restorations (14.7%)	NR	60
Malo et al. 2014	39	100% (*n* = 39)	NR	NR	3 prostheses fracturing in 3 patients. 2 acrylic prostheses fractured at 48 and 58 months of follow-up, whilst a metal ceramic prosthesis fractured at 49 months	NR	Abutment screw loosening occurred in 1 patient at 54 months and prosthetic screw loosening in 2 patients at 47 and 55 months	60
Davo, Malevez and Pons 2013	36	NR	97.2% (*n* = 35	Clinically stable prosthesis that not been removed for two weeks or more during the study period	NR	Replacement of resin teeth in 4 definitive acrylic fixed prostheses after 4 years of function due to extreme tooth wear	NR	60
Branemark et al. 2004	28	82% (*n* = 23)		NR	NR	NR	NR	60
Davo 2009	21	NR	95.8% (*n* = 20)	Clinically stable prosthesis that not been removed for two weeks or more during the study period	NR	NR	NR	60
Agliardi et al. 2017	83	NR	100% (*n* = 83)	In function without mobility or pain, even if one or more of the implants were lost	NR	NR	NR	85.1
Chana et al. 2019	88	93.98% (*n* = 82)	NR	NR	Failure only in the fixed prosthetic group, when compared to the removable prosthetic group (*n* = 5/56 and *n* = 0/27, respectively). Failure was related to 1 fixed acrylic bar and 4 metal acrylic fixed prostheses	NR	Reported loosening in 7 abutments. 1 patient experienced abutment loosening twice within a 12-month period	90
Miglioranca et al. 2012	21	95.2% (*n* = 20)	NR	NR	Fracture of the metal bar supporting a prosthesis in 1 patient	NR	NR	96
Fortin Y. 2017	58	100% (*n* = 58)	NR	NR	NR	NR	NR	100.8
Bothur et al. 2015	14	92.9% (*n* = 13)	NR	NR	NR	NR	Reported loosening of 2 angled abutments and a third with a damaged thread	111.6
Aparicio et al. 2014	22	95.5% (*n* = 21)		NR	4 incidents of fracture of the acrylic coating material, 25 fractures of the porcelain coating material, and fracture of 2 metal frameworks		Reported 6 episodes of screw fractures and 9 episodes of screw or abutment loosening	120

### Sinus pathology

#### Sinusitis

**Fig. 9:**
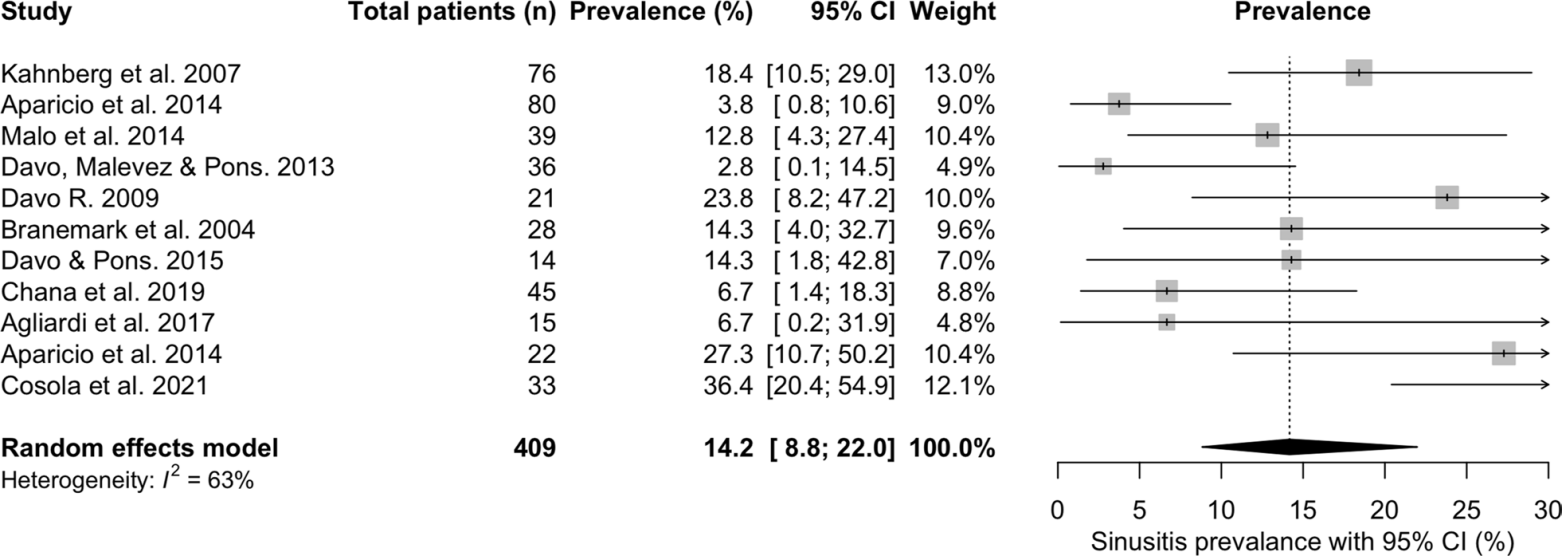
Prevalence of sinusitis (%)

Sinusitis was reported by 11 studies with a total prevalence of 14.2% [95% CI 8.8; 22.0] over a mean of 65.4 months follow-up (Fig. 9). The prevalence of sinusitis ranged from 2.8% [95% CI 0.1;14.5] at 60 months mean follow-up [20], to 36.4% [95% CI 20.4; 54.9] at 141.6 months of mean follow-up [30]. Disease was diagnosed clinically, radiographically, using patient reported questionnaires, or combined methods [11]. Sinusitis was the most commonly reported factor related to implant loss.

Table 7 reports the data involving the sinus within the studies.

**Table 7:** Reported sinus pathology

Study	Mean follow-up	% of patients experiencing sinusitis during follow-up	Individual patient level timeframe data	Presence of oro-antral communication/fistula during follow-up	Intra or extra-sinus zygomatic implant path	Zygomatic implant loss related to sinusitis	Management	Previous history of sinusitis
Kahnberg et al. 2007	36	18.4% (*n* = 14)	Not reported	3.9% (*n* = 3)	Path involving the sinus	Not reported	Not reported	Not reported
Aparicio et al. 2014	55.24	3.7% (*n* = 3)	Not reported	2.5% (*n* = 2)	Path involving the sinus	Not reported	Not reported	Not reported
Malo et al. 2014	60	13% (*n* = 5)	Sinusitis diagnosed in 2 patients at 2 months then individual patients at 6, 12 and 24 months, respectively	1.08% (*n* = 1)	Extra-maxillary approach	1.08% (*n* = 1) in 2.6% (*n* = 1) patient	Antibiotics for 2 patients and surgical management for 2 successful for 4 of 5 patients	13% (*n* = 5) patients
Davo, Malevez and Pons 2013	60	1.7% (*n* = 1)	Not reported	Sinusitis (*n* = 1) Pain and swelling at zygomatic level (*n* = 1)	Intra and extra-maxillary paths	Not reported	Antibiotics and meatotomy successful	Not reported
Davo 2009	60	23.8% (*n* = 5)	Not reported	Not reported	Path involving the sinus	No failures due to sinusitis	2 patients Antibiotics, 2 patients meatotomy or 1 patient Caldwell Luc. All successful treatments	Not reported
Branemark et al. 2004	60	14.2% (*n* = 4)	Not reported	Not reported	Path involving the sinus	Not reported	Meatotomy on 4 patients	3.5% (*n* = 1) patient
Davo and Pons 2015	60	14% (*n* = 2)	Sinus infection diagnosed at 24 and 30 months, respectively	Fistula at 1 implant	Path involving the sinus	Not reported	Antimicrobial therapy successful in managing disease	Not reported
Chana et al. 2019	90	6.6% (*n* = 3)	Not reported	Not reported	Intra and extra-maxillary paths	3.4% (*n* = 3) in 6.6% (*n* = 3) patients	Not reported	Not reported
Agliardi et al. 2017	85.04	6.7% (*n* = 1)	5 months after placement	6.7% (*n* = 1)	Path involving the sinus	0% (*n* = 0)	Antimicrobial therapy successful in managing disease	Not reported
Aparicio et al. 2014	120	27.3% (*n* = 6)	Post placement, 1–2 years, 2–3 years, 7–8, 8–9, 10–11	3.75% (*n* = 80)	Path involving the sinus	1.5% (*n* = 2) in 4.5% (*n* = 1) patient	5 patients successfully managed with antimicrobials	Not reported
Cosola et al. 2021	141.6	32.8% (*n* = 12)	Not reported	Not reported	70.2% (*n* = 47) completely intrasinusal. 29.85% (*n* = 20) not completely intrasinusal	23.9% (*n* = 16) in 24.24% (*n* = 8) patients	Not reported	Not reported

### Oral health impact of zygomatic implant therapy

Patient reported outcomes using the Oral Health Impact 14 score (OHIP14) were reported by Pellegrino et al. [16] and Davo and Pons [18]. Pellegrino et al. [16] reported OHIP scores of 30.4 (± 9.5) pre-operative and 6.3 (± 3.7) after 60 months follow-up. Davo and Pons [18] reported total mean OHIP14 scores of 3.4, 2.5, 3.8 at 1, 2 and 5 year follow-up recalls, respectively. No pre-operative OHIP14 questionnaires were carried out.

Oral Health Impact Profile Edentulous questionnaires (OHIP EDENT) were reported for 22 patients within a control group (Classical Branemark approach) [29]. 84% reported satisfaction scores above 80%. 31.8% of those patients reporting a maximum satisfaction score of 100%. This control group was compared against a test group (ZAGA approach), of which 76.3% (*n* = 61) recorded satisfaction rates of between 81 and 100% [11]. The difference between the groups was not statistically significant (*P* = 0.92).

A Likert scale to investigate satisfaction following a full-arch, immediate restoration supported by ZIs, was reported by Agliardi et al. [13]. Aesthetics and function were reported as excellent or very good by the entire group of 15 patients. Phonetics was considered excellent or very good by 13 of 15 patients.

Subjective satisfaction for oral rehabilitation using ZIs and prosthetic reconstructions were reported by Kahnberg et al. [15]. 73 Patients reported 86% satisfaction with the aesthetics and 71% satisfaction with the functional outcomes at 3 years of follow-up.

Malo et al. [19] assessed aesthetic and functional complaints in a group of 39 patients over a 5-year period. There were no reports of complaints at the final follow-up by any patients.

### Assessment of bias

The risk of bias assessment is summarised in Table 8. Consecutive inclusion of participants and complete inclusion of participants presented the most common risk of bias, as this was unclear in several studies. Older studies reported data in a less systematic fashion when compared to contemporary studies. Descriptive analysis was the most common reporting methodology.

**Table 8:** Assessment of studies using the Joanna Briggs Institute critical appraisal tools for case series

Study	Was there clear criteria for inclusion?	Was the condition measured in a standard, reliable way for all participants?	Were valid methods used for identification of the condition for all participants included in the case series	Did the case series have consecutive inclusion of the participants	Did the case series have complete inclusion of participants	Was there clear reporting of the demographics of the participants in the study	Was the clear reporting of clinical information of the participants	With the outcomes or follow-up results of cases clearly reported	Was there clear reporting of the presenting site/clinic demographic information	Was statistical analysis appropriate
Kahnberg et al. 2007	Unclear	Unclear	Unclear	Unclear	Unclear	Unclear	Yes	Yes	Yes	Not applicable
Pellegrino et al. 2020	Yes	Yes	Yes	Unclear	Unclear	Yes	Yes	Yes	Unclear	Yes
Aparicio et al. 2014	Unclear	Yes	Unclear	Yes	Unclear	Yes	Yes	Yes	Yes	Not applicable
Coppede et al. 2017	Yes	Yes	Yes	Unclear	Unclear	Yes	Yes	Yes	Yes	Not applicable
Davo & Pons. 2015	Yes	Yes	Yes	Yes	Yes	Unclear	Yes	Yes	No	Not applicable
Malo et al. 2014	Yes	Yes	Yes	Unclear	Unclear	Yes	Yes	Yes	Yes	Not applicable
Davo, Malevez and Pons 2013	Yes	Yes	Yes	Yes	Unclear	Unclear	Yes	Yes	Yes	Not applicable
Davo 2009	Yes	Yes	Unclear	Yes	Unclear	Unclear	Yes	Yes	Yes	Not applicable
Branemark et al. 2004	Unclear	Unclear	Unclear	Yes	Unclear	Yes	Yes	Yes	Yes	Not applicable
Yates et al. 2013	Yes	Yes	Yes	Unclear	Yes	Unclear	Yes	Yes	Yes	Yes
Bedrossian 2010	Yes	Yes	Yes	No	No	No	Unclear	Yes	No	Not applicable
Agliardi et al. 2017	Yes	Yes	Yes	Yes	Unclear	Yes	Yes	Yes	Yes	Not applicable
Chana et al. 2019	Yes	Yes	Yes	Unclear	Yes	Yes	Yes	Yes	Yes	Not applicable
Miglioranca et al*.* 2012	Yes	Yes	Yes	Unclear	Unclear	Yes	Yes	Yes	Yes	Not applicable
Fortin 2017	Unclear	Yes	Yes	Yes	Unclear	Unclear	Yes	Yes	Yes	Not applicable
Bothur et al*.* 2015	Unclear	Yes	Yes	Unclear	Unclear	Yes	Yes	Yes	Yes	Not applicable
Aparicio et al. 2014	Yes	Yes	Yes	Yes	Unclear	Yes	Yes	Yes	Yes	Yes
Cosola et al. 2021	Yes	Yes	Yes	Unclear	Unclear	Yes	Yes	Yes	Yes	Yes

## Discussion

ZIs present a therapeutic opportunity to rehabilitate patients who lack either the desire to undergo extensive augmentation procedures, or lack the anatomical structures required, to deliver conventional implant therapy in the maxilla. Custom, increased-length, end osseous implants anchored in the zygomatic process was first reported by Aparicio et al. in 1993 [2], with subsequent international studies exploring this treatment modality and its outcomes. The present systematic review focused on long-term ZI survival (≥ 3 years). Unfortunately no controlled trials, randomised or otherwise, that met our criteria were identified. Severe maxillary atrophy was the predominant clinical indication, and described patients who were unable to receive conventional implants without additional augmentation procedures. Other clinical indications for ZIs included trauma, cleft, and oncologic patient groups. There was a paucity of studies meeting the inclusion criteria for ZI placement in oncology patients. This could be related to the reduced survival rates of such individuals, alongside the increased risk of osteoradionecrosis (ORN) for those who received adjuvant radiotherapy to the zygomatic region. Only one study within this systematic review [16] compared ZI survival in the atrophic maxilla against ZIs placed after oncologic resections, and identified no difference in survival between the groups.

### Zygomatic implant survival

In this review, ZI survival was 96.2% [95% CI 93.8; 97.7] over the mean follow-up of 75.4 months (6.3 years). The results are consistent with two other systematic reviews. The first included 68 studies [32], which reported a cumulative survival rate of 95.2% with no minimum follow-up, to a maximum follow-up of 12 years. The second, Sola Perez et al. [33] reported survival rates of 98.5% within the first year, 97.5% between 1 and 3 years, 96.8% at 3–5 years and 96.1% at more than 5 years. The current study also investigated the overall incidence of ZI failure (failure being defined as the antagonist of ZI survival), incidence of failure within the first year, and incidence of failure in subsequent years. When ZI failure did occur, sinusitis, oro-antral communications, failure of osseointegration, loss of osseointegration, infection or peri-implantitis, pain and zygomatic implant fracture were the related factors in descending order of frequency. A higher incidence of failure was identified within the first year (2%) compared to that of subsequent years (0.5%/year), whilst the overall annual failure incidence was 0.7% with intra-study mean follow-up times ranging from 36 to 142 months, again corroborated by the systematic review from Chrcanovic et al. [34], and Chrcanovic et al. [32]. These findings reflect the possibility that some patients will suffer from early infective, or medically related, post-surgical complications, which may result in ZI loss.

We found the mean ZI survival rate to be higher for immediate Loading protocols over delayed loading protocols. Di Cosola et al. [30] reported that immediate loading resulted in significantly lower risk of infective, neurological and overall complications compared with the two-step rehabilitation. The latter finding was supported by Chrcanovic et al. [32], who hypothesised this to be related to the length of follow-up, as delayed loading protocols were associated with increased time frames allowing greater chance of failure to occur during this period. The slightly higher ZI survival rate could also be due to evolution in the delivery of ZI therapy. Delayed load protocols are associated with the original technical approach, whilst immediate load protocols were introduced alongside refinements to the surgical technique, operator familiarity with the procedure, and ZI/ZI reconstruction design innovations. The difference was statistically significant between the protocols (*p* = 0.03) but may not be clinically relevant, and so should be interpreted with caution. Both loading protocols result in high ZI survival prevalence.

A common theme across all studies, was the combination of ZI in the posterior maxilla splinted to conventional implants in the anterior maxilla. Moraschini et al. [35], through a systematic review, compared ZI survival to conventional implant survival, which reported survival at 96.5% ± 5.0 for ZIs and 95.8% ± 6.4% for conventional implants at 78 months of mean follow-up. These findings suggest that ZIs have long-term survival rates comparable to conventional implants, with a positive inference for the ability to deliver dual implant modality supported reconstructions.

ZI survival rates also appear to be comparable to alternative techniques in the atrophic maxilla, including short implants, tilted implants, and implants placed in grafted sinuses. Slot et al. [36] reported 96.1% and 100% survival at 10 years in a randomised controlled trial of 6 or 4 conventional implants, respectively, when supporting maxillary overdentures. A meta-analysis and systematic review by Kotsovilis et al. [37] revealed no statistically significant difference in survival between short (≤ 8 mm or < 10 mm) and conventional (≥ 10 mm) rough surface implants placed in partially or totally edentulous individuals. ZIs may confer treatment time benefits to patients due to the immediacy of reconstruction when compared to implants placed in grafted sites. Further benefits are realised through reduced morbidity, due to the absence of a second donor sites, and potential cost savings due to a reduced number of procedures or number of conventional implant required to support full-arch prostheses. However, ZI placement is an advanced surgical procedure, requiring appropriate technical skillsets, and may also require sedation or a general anaesthetic, which in turn, adds additional cost and complexity to the process.

### Zygomatic implant success

ZI success was 96%. Reporting ZI success demonstrated challenges because of significant heterogeneity (*I*^2^=90%) across the studies. ZIs in situ was a prerequisite, whilst specifying an absence of pain [13, 16, 17, 19, 20, 26] may have reflected reports of pain and neurosensory disturbances in the zygomaticofacial and infraorbital regions reported in other studies [21, 24, 25]. Absence of infection (included sinus disease, oro-antral communications, peri-implantitis and peri-implant mucositis) was included by 3 studies [13, 19, 23]. There are no recognised criteria for ZI success, and criteria for conventional implant success, as reported by Albrektsson and Isidor [38], have been suggested as a reference. Radiographic changes were considered as success criteria for 3 studies [13, 19, 21]. 3-dimensional imaging may provide the most accurate data on bone volume maintenance, but is unsuitable as a tool for monitoring success due to the dose of radiation received by the patient. Plain film, 2-dimensional, radiographic examinations are unsatisfactory for monitoring bone volume around ZIs, due to anatomical challenges and the possible fixture head positions in relation to the alveolar crest [39]. An attempt to standardise these reporting challenges has been suggested by Aparicio et al. [40] with the publication of the ORIS criteria (offset of prosthesis, rhino-sinusitis status, infection in soft tissue, stability of ZI) for documenting ZI success.

### Zygomatic prosthesis survival and success

Prosthesis survival ranged from 82 to 100% with the mean at 94% at 76 months of follow-up. The high prosthesis survival mirrored that of ZI survival, although there was a potential for confounding as the definitive prosthesis was placed between 3 and 6 months after ZI installation. Early ZI failures could have influenced survival of the provisional prosthesis, but not the definitive reconstruction. Provisional prosthesis success was not reported in the literature, but there was little mention of complication rates as a counter argument. The mechanism of catastrophic failure was either related to loss of the supporting ZI or conventional implants, or fracture of the reconstruction [11, 18, 19, 25, 26]. Fracture of the metal substructure or fracture of the ceramic / acrylic attached to the metal substructure were the reported modes of prosthesis failure. Although not reported in this study, reconstructions supported by conventional implants alone showed no statistically significance difference between the construction materials used for full-arch screw-retained prostheses [41]. Mean ZI prosthesis survival appears comparable to full-arch screw-retained fixed prostheses supported by conventional implants. Sailer et al. [42] reported a 5-year cumulative survival of 95.8% for full-arch, screw-retained prostheses, whilst Wittneben et al. [41] reported a 5-year cumulative survival of 96.7% in comparison to this review, with 94% survival at 6 years for combined full-arch and partial ZI-supported reconstruction data.

Significant heterogeneity was found when reporting prosthesis success. Success criteria, such as allowing a prosthesis to be out of the mouth for up to 2 weeks, potentially affected the results and allowed for a higher prosthesis survival rate. In addition, Kahnberg et al. [15] and Branemark et al. [22], when faced with ZI loss, reported modification rather than replacement of the definitive prosthesis. The use of acrylic/acrylic teeth across the majority of studies potentially aided survival rates, as the prosthesis was repairable rather than lost. Chipping of the veneering resin was reported to be the second most common complication after loss of retention [41]. Technical complications for full-arch ZI screw-retained reconstructions appear to reflect those seen in conventional full-arch screw-retained implant reconstructions. Screw and abutment loosening events were frequently reported [11, 15, 16, 18, 19, 25, 28]. These complications have been reported in the implant literature [42], but adverse incidence rates for ZI reconstructions could be influenced by increased movement of the prosthesis due to bending moments of the ZIs [18, 20, 28]. These technical complications occurred regardless of whether the ZIs were splinted to conventional implants, or whether the prosthesis was supported by Quad ZIs. It is also possible that this bending phenomenon contributed to veneering material fractures away from the metal substructure. If a similarity between ZI-supported and conventional implant-supported prostheses is considered, Sailer et al.’s [42] report suggests that patient education and consent is essential in managing patients’ expectations when a 54.1% technical complication rate over 5 years exists for screw-retained, full-arch, conventional implant-supported prostheses.

### Sinus pathology

The overall prevalence of sinusitis was 14.2% with a follow-up of 65.4 months. There was significant heterogeneity in the methodology employed to diagnose sinusitis across the studies, which included clinical, radiographic, patient reported questionnaires, or combined methods [11]. The 95% CI ranged from 8.8 to 22%, with an increased prevalence over longer follow-up periods [20, 30]. This finding might suggest a relationship between the two, although a reported confounding factor is the background population prevalence of chronic rhinosinusitis. This has been reported at > 10% in a western population, when measured by objective criteria [43], but when using guideline-based diagnostic criteria, the true prevalence of chronic rhinosinusitis is reportedly less than 5%.

The classical approach, or Branemark protocol [22], for ZI placement is via an intra-sinus route, with a window raised in the lateral sinus wall to allow direct visualisation for ZI placement. In contrast, the extra-sinus  approach, first reported by Miglioranca [51] does not breach the sinus cavity during placement although the extra-maxillary approach may do so at the at the most apical extent before entering the Zygoma. Extra-maxillary refers to the coronal portion of the implant at intra-oral exit site. The biological consequences of the presence or absence of a ZI within the sinus cavity are therefore worth consideration. Sinusitis was the most commonly reported factor related to ZI loss within this review. Aparicio et al. [11] reported a statistically significant difference in Lund–MacKay sinus staging score (radiographic examination) between classical and ZAGA groups (*p* = 0.04). Dual Lanza–Kennedy scores (patient reported) and Lund–MacKay scores, again reported a statistically significant difference between the treatment protocols. Sinus pneumatisation was found to be statistically related to overall complications and implant loss by Di Cosola et al. [30] However, these findings may relate to the fact that larger sinuses require an intra-sinus path for ZI placement, rather than presenting the surgical option for an extra-maxillary approach. It would appear logical that ZIs not breaching the sinus wall would not be likely to cause sinusitis. Ultimately, the patient’s individual anatomy and the desired prosthetic envelope, dictate the ZIs trajectory towards an intra- or extra-sinus approach.

When sinusitis was diagnosed, successful treatment with antibiotics and/or via a surgical meatotomy was reported with no further consequences. ZI failure due to loss of osseointegration, was infrequent when presented on a background of sinusitis, with surgical removal being the more common mode of ZI loss to treat un-resolving sinusitis. This suggests sinusitis may not always be a catastrophic event, although a concern of bacterial colonisation onto an exposed ZI surface within the sinus, with subsequent inflammation or infection might be prudent. Petruson [44] conducted sinuscopies on patients whose ZIs had been in function after at least 1 year. Total or partial mucosal coverage had occurred with no signs of infection or increased secretion. However, there is potential evidence to show that inflammatory bone changes may occur, as Bothur et al. [28] reported signs of osteitis when examining sinus walls (not directly surgically altered) after ZI placement. New bone development, measured radiographically, was seen in all patients within at least one of their sinuses. Di Cosola et al. [30] reported that a sinus mucosa thickness of > 3 mm was related to an increased odds ratio (1:2.8) of infective complications (sinusitis, oro-antral fistula, infection of the soft tissues). Obstruction of the osteum was not associated with implant failure or infective complications. Davo et al. reported no clinical consequences in patients who exhibited radiological thickening of the sinus mucosa associated with zygomatic implants [45]. It appears that ZIs within the sinus are not always a cause of clinically diagnosed or reported sinusitis, and that antibiotic therapy or surgery can successfully treat acute cases of sinusitis. Some cases may persist however, with a need to remove the ZI in order to resolve the situation.

Oro-antral communication was also linked to ZI loss [11, 13, 15, 19, 20]. Preventing oro-antral communications is reliant on hard or soft tissue attachment to the coronal aspect of the implant. It has been suggested that 2 stage procedures, with repeat surgery or abutment changes at this level, could have a negative effect [7]. Early infective processes may also jeopardise this seal, as potentially might bending movements when in function. Patient education, compliance in oral hygiene measures, and prosthesis cleansability are undoubtedly important in maintaining peri-ZI tissue health and for the long-term prevention of peri-ZI mucositis.

### Patient reported outcomes with zygomatic implants

Across the studies, there was significant heterogeneity in recording patient reported outcomes. Few studies investigated PROMs with the same tool, which made comparison between studies impossible. PROMs where investigated via the OHIP 14 system [16, 18] developed by Slade et al. [46], the OHIP EDENT [11] or via Likert-style questionnaires [13]. Improvements were reported in all studies comparing qualify of life (QoL) start points to end points. When considering conventional implant literature and associated reconstructions, edentulous patients report improvements in satisfaction when provided with implant retained or supported prostheses, regardless of fixed or removable design. Removable was found to be preferential for performing hygiene related procedures [47]. In addition, either immediate or delayed reconstructions were found to be acceptable. Heydecke et al. [48], in a crossover study, found implant retained removable prostheses to be preferred over fixed reconstructions for phonetics. This was either due to prior patient experience with palatal coverage, or that not enough time was given for adaptation. An extended period for normalisation might therefore be recommended. Conversely, Brennan et al. [49], reported patients with fixed reconstructions were more satisfied than counterparts with removable reconstructions. Edentulism is recognised as having a detractive effect on the emotional status of patients [50]. Any implant-supported or retained reconstruction might reasonably be expected to improve QoL if individuals previously managed with conventional removable prostheses, or had undergone partial or total maxillary resection for trauma or oncology. Factors influencing satisfaction were comfort, aesthetics and phonetics. These are more challenging in ZI therapy, which may have a more palatal emergence profile of the implant platform related to the local anatomy and level of alveolar bone atrophy. This may lead to prostheses that encroach upon the palate, and might challenge patients’ adaptive capabilities. This relies on surgical skill coupled with prosthetically driven planning to reduce the chance of prosthetic complications. The use of PROMs and QoL assessments within ZI research is an essential component of patient-centred research, but standardised criteria and recording by investigators is recommended to construct a more detailed picture.

### Limitations of the evidence

Limitations centred around the quality of reporting. Although the inclusion criteria of primary studies were clear, it was often unclear as to whether there had been consecutive and/or complete inclusion of the participants. As such, inclusion bias may have played a part in the selection of participants selected for studies. Older studies were less systematic in their approach to reporting. Patient demographics were generally well reported. Narrative results were sufficient to extract datasets that allowed the systematic review to be conducted along with the meta-analysis. Heterogeneity in reporting ZI success and PROMs was notable across the studies, which challenged comparisons. However, we tried to account for several factors that led to heterogeneity, e.g., the duration of follow-up (i.e. by calculating annual incidence rates) and loading protocol (i.e. by performing subgroup analysis).

### Limitations of the review

This systematic review followed the PRISMA guidelines for reporting, which created a framework for the structure, assessment, introspection, and reporting process in order to conduct a review and analysis of the relevant literature. The main limitations were related to the study quality, as no clinical trials or randomised controlled trials were included. In addition, we were unable to analyse ZI success for all studies, or carry out further subgroup analyses of complications due to heterogeneity in reporting. Caution should be used when interpreting the results due to the lack of high-quality evidence.

## Implications for practice and future research

These results indicate that ZIs are a predictable treatment modality for use in the atrophic maxilla, and represent a reconstructive therapy to consider against techniques including sinus augmentation with conventional implant rehabilitation. Five-year ZI survival rates appear comparable to conventional implants, although mean longer term comparable survival and complication data are still lacking. The reported survival rates are reassuring, as ZI therapy presents the opportunity for immediate reconstruction at the time of implant placement, and reduces both morbidity from donor site procedures and reduces overall treatment times. The study indicates that ZI-supported prosthetic reconstruction survival is satisfactory, with complications comparable to that experienced by reconstructions supported by conventional implants. Further investigation into ZI-supported prosthesis performance, material choices and prosthetic complications are required. Sinusitis appears to be a complication affecting around 14% of individuals with ZIs, but can respond to antibiotic or surgical treatment with total resolution. PROMs indicate that ZIs improve quality of life for those treated in this fashion.

Future research should be focused on the creation of uniform research core datasets to ensure standardised reporting within the field of ZIs, in order to capture and compare study data. There is also a need to identify guidelines for diagnosis and management of sinus pathology related to ZIs. Finally, standardised PROMs should be included when measuring clinical outcomes.

## Conclusions

Zygomatic implants may represent a predictable treatment modality for management of the atrophic or resected maxilla, with comparable survival rates to conventional implants over similar time frames. Immediate loading showed a statistically significant increase in survival rates over delayed loading, but this difference may not be clinically significant. Prosthesis survival was satisfactory and similar to that of prostheses supported by conventional implants, with similar complications. Sinusitis was the most frequently encountered biological complication. Patient reported outcomes show an increase in satisfaction when rehabilitated with ZIs.

## Supplementary Information

**Additional file 1: Table S1: **Search strategy.** Table S2: **Excluded studies sorted according to the reason of exclusion after full-text screening.

## Data Availability

All data generated or analysed during the study are included in this published article.

## Abbreviations

ZI: Zygomatic implant
PROM: Patient reported outcome measures
PRISMA: Preferred Reporting Items for Systematic Review and Meta-Analyses
PROSPERO: Prospective Register of Systematic Reviews
PIO: Patient, intervention, outcome
OHIP: Oral health impact profile
OHIP EDENT: Oral health impact profile edentulous
QoL: Quality of life
CI: Confidence interval
ZAGA: Zygoma anatomy guided approach
ORIS: Offset, rhino-sinus, infection, stability
NR: Not reported
N: Number
