# Supplementary material for: Long-term treatment outcomes with zygomatic implants: a systematic review and meta-analysis
Source: Int J Implant Dent. 2023 Jul 5;9:21. doi: 10.1186/s40729-023-00479-x (PMC10322814; doi:10.1186/s40729-023-00479-x)
Supplement: Supplementary file 1 — Additional file 1: Table S1: Search strategy. Table S2: Excluded studies sorted according to the reason of exclusion after full-text screening. [file 40729_2023_479_MOESM1_ESM.docx]

Additional file 1

**Table S1:** Search strategy.


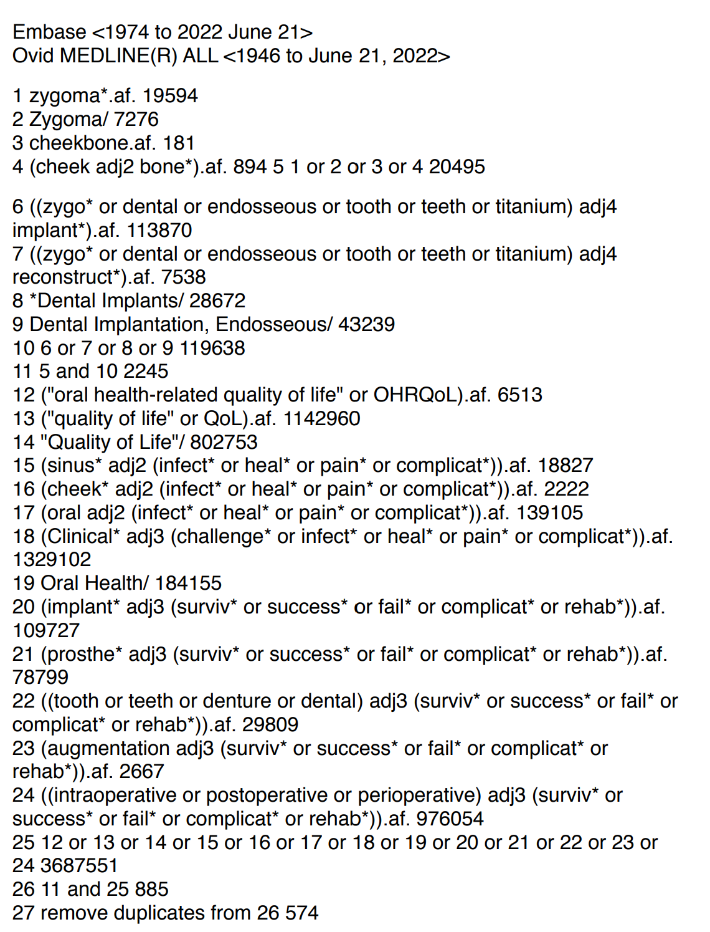


**Table S2:** Excluded studies sorted according to the reason of exclusion after full-text screening (n=37)

| **Reason for exclusion** | **Author** |
| --- | --- |
| JBI checklist exclusion | Petrungaro et al. 2019  Alexsandrowicz et al. 2019 |
| < 3 years follow-up | Agliardi et al. 2020  Alberto et al. 2021  D’Agostino et al. 2021  Padovan et al. 2021  Carvalho et al. 2021  Butterworth. 2018  Wang et al. 2015  Araujo et al. 2017  Balan et al. 2017  Neugarten et al. 2017  Agbara et al. 2017  D’Agostino et al. 2016  Lombardo et al. 2016  De Araujo Nobre et al. 2015  Sartori et al. 2012  Miglioranca et al. 2011  Zwahlen et al. 2006 |
| Follow-up range not reported | Aleksandrowicz et al. 2020  Balshi et al.2012 |
| < 10 patients | Goker et al. 2020  Landes et al. 2013  Grecchi et al. 2021  Zou et al. 2014 |
| Same patient group reported in another study | Davo R. 2013  Malo P.2012 |
| Not using zygomatic implants | Alresayes et al. 2022  Chrcanovic et al. 2017 |
| Zygomatic implant survival data not fully reported | Mahendran et al. 2022  Huang et al. 2021  Aleksandrowicz et al.2020  Almedia et al. 2017  Alzoubi et al.2017 |
| Abstract only | Dibirov, Drobyshev. 2019  Guerra et al. 2013 |
| Review paper | Pommer et al. 2014 |
